# Moderating Effect of Race and Ethnicity Between Technology Use and Social Isolation

**DOI:** 10.1093/geroni/igaa057.333

**Published:** 2020-12-16

**Authors:** Sunghwan Cho, Tommy Buckley, Kyeongmo Kim

**Affiliations:** Virginia Commonwealth University, Richmond, Virginia, United States

## Abstract

Social isolation among older adults has brought about poor outcomes of their health and well-being. Information and Communication technology (ICT) is known to alleviate social isolation of older adults. However, it is unknown how ICT access and use are associated with social isolation by race. This study examined the association of ICT and social isolation from the National Health and Aging Trends Study (NHATS), estimating moderating effects of race and ethnicity. The sample for this study was community dwelling Medicare beneficiaries aged 65+ (n=5,567). An index for ICT was formed from five domains: social network websites, email and texting, working cellphone, tablet devices and online computer use (range 1-5, mean=3.96, SD=0.927), and social isolation was derived from responses to five areas: living arrangement, attending religious activities, numbers of important people to talk with, attending other activities such as club participation and volunteer work (range=0-2, mean=1.74, SD=0.927). Race and ethnicity included White (69.6%), African-American (20.7%), Hispanic (5.5%) and Asian/Pacific Islander (2.5%). Multiple linear regression was used using a moderating effect of race/ethnicity, including relevant covariates. Findings revealed increased ICT use was associated with lower social isolation (b=0.05, p<0.05), and race/ethnicity was a significant moderator in the association between ICT and social isolation for African-Americans (b=0.08, p<0.05) and Hispanics (b=0.15, p<0.05) compared to White older adults. The findings indicate that racial differences should be considered when applying technology use to reduce older adults’ social isolation. Practitioners can provide racially competent ICT services for older adults interested in tech-based communication.

